# Salt and cardiovascular disease in PURE: A large sample size cannot make up for erroneous estimations

**DOI:** 10.1177/1470320318810015

**Published:** 2018-11-08

**Authors:** Monique Tan, Feng J He, Graham A MacGregor

**Affiliations:** Wolfson Institute of Preventive Medicine, Barts and The London School of Medicine & Dentistry, Queen Mary University of London, UK

**Keywords:** Salt, cardiovascular disease, mortality, salt reduction programmes, prevention

## Abstract

The latest Prospective Urban Rural Epidemiology (PURE) study claims that salt reduction should be confined to settings where its intake exceeds 12.7 g/day and that eating less than 11.1 g/day of salt could increase cardiovascular risk. More specifically, Mente et al. suggested that (a) salt intake was positively associated with stroke only when it exceeded 12.7 g/day, (b) salt intake was inversely associated with myocardial infarction and total mortality, and (c) these associations were largely independent of blood pressure.

These provocative findings challenge the robust evidence on the role of salt reduction in the prevention of cardiovascular disease and call into question the World Health Organization’s global recommendation to reduce salt intake to less than 5 g/day.

However, Mente et al.’s re-analysis of the PURE data has several severe methodological problems, including erroneous estimations of salt intake from a single spot urine using the problematic Kawasaki formula. As such, these implausible results cannot be used to refute the strong evidence supporting the benefits of salt reduction for the general population worldwide.

The latest Prospective Urban Rural Epidemiology (PURE) study^1^ has stirred controversy and confusion in the popular press and the general public. Its findings are ‘exceedingly provocative’^2^: a strong association between salt and stroke was seen only in the highest tertile of salt intake (in this study, over 12.7 g/day) and not at lower intakes, and salt was also found to be inversely associated with myocardial infarction and total mortality, thus implying that the lowest tertile of salt intake (below 11.1 g/day) could be harmful (i.e. a J-shaped relationship). Potassium intake, on the other hand, had inverse associations with all cardiovascular outcomes. These associations were said to be largely independent of blood pressure (BP).^1^

These controversial findings result from a re-analysis^3^ of data collected in individuals aged 35–70 years from the general population of 18 countries, with no history of cardiovascular disease (CVD) at recruitment. The association between salt intake and BP used data from 95,767 participants (in 369 communities) and the data on CVD were from 82,544 participants (in 255 communities). All analyses were performed at the community level, but adjustments were made with individual-level data. A community was defined as a group of people sharing characteristics and living in defined geographical areas.^1^

The size and scope of this work are impressive, but still cannot make up for the fact that the participants’ salt intake was estimated from a single spot urine (here, a morning midstream urine sample) using the Kawasaki formula.^4^ The unreliability and systematic bias of this method has been demonstrated in many studies,^5–8^ irrespective of collection timing: whether it is collected in the morning, afternoon, evening or overnight, a spot urine systematically yields biased estimates of salt intake.^9^

The issue may also, at least partially, lie in the Kawasaki formula^4^ itself:

$$24h\;\mathrm{UNa}\;\left[\frac{mmol}{day}\right]=\;16.3\times \;\sqrt{\frac{spot\;Na\;\left[\frac{mmol}{L}\right]}{spot\;Cr\;\left[\frac{mg}{dL}\right]\;\times \;10}\times \;Predicted\;24h\;UCr\;\left[\frac{mg}{day}\right]}$$

where 24h UNa = 24-hour urinary sodium, spot Na = sodium concentration from spot urine, spot Cr = creatinine concentration from spot urine and 24h UCr = 24-hour urinary creatinine, and where the predicted urinary creatinine for females is as follows: creatinine [mg/day] = (**−**4.72 × age [years]) + (8.58 × weight [kg]) + (5.09 × height [cm]) − 74.5; and for males is as follows: creatinine [mg/day] = (−12.63 × age [years]) + (15.12 × weight [kg]) + (7.39 × height [cm]) − 79.9.

One can see that the formula relies on several variables, including sex, age, weight, height and creatinine concentration. The problem is that most of these parameters are highly correlated with health outcomes, particularly age, which is the biggest risk factor for death. When looking at the relationship between salt intake and CVD risk, to estimate the former using equation parameters linked to the latter raises serious issues of confounding. In other words, due to the inherent nature of the Kawasaki formula, the association between salt intake and CVD risk is impossible to distinguish from that between the equation parameters (sex, age, weight, height and creatinine) and CVD risk.

The most reliable way to assess an individual’s current salt intake is to collect urine over 24 hours, and to repeat this several times: studies have shown that at least three 24-hour urine collections are needed to get a reasonably reliable estimate of one’s current salt intake, due to large day-to-day variations.^10^ So far, only three cohorts have deployed this gold standard method and all have shown a direct, linear relationship between salt intake and CVD risk, as well as mortality,^7,11–13^ down to a salt level of 3 g/day.^7^

A head-to-head comparison of the two methods for salt intake assessment and their association with mortality has been made using data from the Trials of Hypertension Prevention (TOHP).^7^ In the first analysis, salt intake was assessed by averaging three to seven 24-hour urinary sodium excretions (i.e. gold standard method); this produced a direct and linear relationship between salt intake and all-cause mortality. In a second analysis, salt intake was estimated from sodium concentrations using the Kawasaki formula; this altered the relationship and made it appear J-shaped. Going one step further, the researchers demonstrated the importance of multiple 24-hour urine collections by repeating the exercise, but this time using only the first 24-hour urine collected instead of averaging three to seven collections; again, the shape and direction of the association were altered, regardless of the estimation method (Figure 1).^7^ This clearly shows that estimating salt intake from sodium concentrations by the Kawasaki formula produces an erroneous J-shaped relationship with mortality and that one single measure cannot reflect an individual’s usual salt intake, the accurate assessment of which is vital for studies associating salt intake with health outcomes.^14^

**Figure 1.**
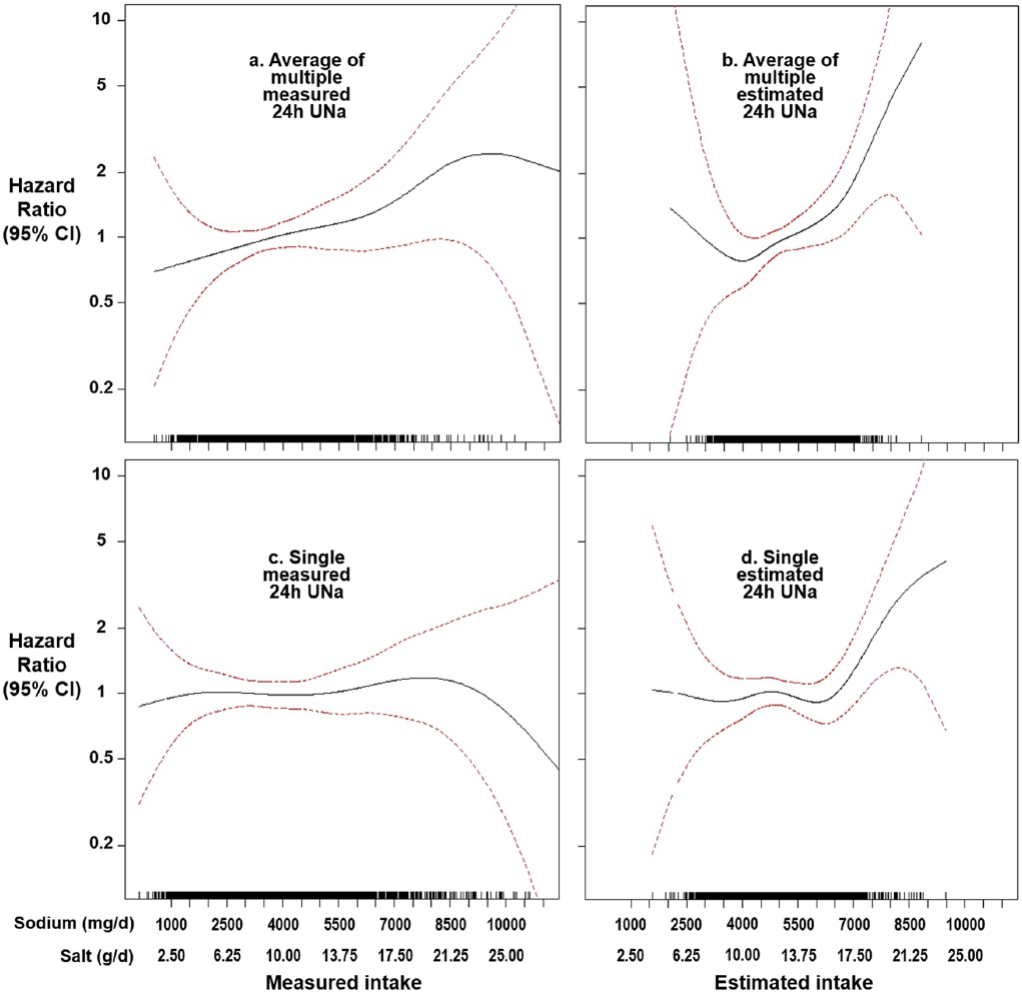
Spline plots on the association between salt intake and all-cause mortality in 2974 individuals who took part in the Trials of Hypertension Prevention, but were not in the salt reduction intervention group, followed up for over 20 years. (a) Salt intake assessed by the average of 3–7 24-hour urinary sodium excretions (i.e. gold standard method) during the Trials of Hypertension Prevention trial period of 18 months to 4 years. (b) Salt intake calculated as the average of 3–7 estimated 24-hour urinary sodium excretions from sodium concentrations using the Kawasaki formula. (c) Salt intake assessed by the first measured 24-hour urinary sodium excretions at baseline of Trials of Hypertension Prevention trials. (d) Salt intake assessed by the estimated 24-hour urinary sodium excretions from the first 24-hour urinary sodium concentration using the Kawasaki formula. 24h UNa: 24-hour urinary sodium excretions.

In an attempt to overcome those methodological challenges, Mente et al performed community-level analyses.^1^ However, such analyses cannot safeguard against the unreliability of weak estimation methods for salt intake. The Kawasaki formula systematically overestimates salt intake at lower intake levels and underestimates it at higher intake levels, and this bias still exists at the population level; in the TOHP for example, the Kawasaki formula overestimated the mean population-level salt intake by 3.2 g/day.^7^ In view of this, it is unclear why Mente et al did not correct for the over- and underestimations.

Another major limitation of the PURE study is that changes in salt intake over time were not taken into account: urine collection took place at baseline and was not repeated afterwards. Participants were enrolled in the study between 2003 and 2013, Mente et al’s analysis included outcome events until September 2017 and the median follow-up time was 8.1 years^1^: can salt intake be assumed to have remained stable all this time? We believe not, since national salt reduction strategies have started in many countries during this time period.^15^

Capturing the fluctuations in salt intake over time is indispensable when examining long-term associations with health outcomes. This was demonstrated in a study where researchers compared the use of a single baseline versus multi-year 24-hour urinary sodium excretions to estimate long-term associations with CVD and renal disease risk. Since the baseline estimates could not account for the important changes in salt intake over the study period (fluctuations exceeded 2.0 g/day in half of the participants), the hazard ratios changed up to 85% when 1- and 5-year follow-up salt intakes were used instead of the baseline values. When using the single baseline estimates only, participants in the highest tertile of salt intake (over 10.8 g/day) had the same CVD risk as those in the lowest tertile (below 7.5 g/day), but repeating this analysis with multi-year estimates yielded a 73–80% increase in CVD risk for the participants in the highest tertile of salt intake.^13^ This shows that a single baseline 24-hour urine collection is inaccurate and imprecise in estimating individuals’ long-term salt intake, and thus insufficient to study the long-term relationship between salt intake and CVD risk. The fact that the baseline salt estimates used by Mente et al were obtained by spot urine and the Kawasaki formula only makes their reliability go from bad to worse.

Furthermore, it is impossible to rule out residual confounding in observational studies. In Mente et al’s latest paper, it is unclear whether diseases other than CVD were taken into account in the adjustments and sensitivity analyses.

Mente et al’s previous publication on salt intake and CVD^3^ gave rise to much criticism, which they failed to address and discuss in their latest paper. For example, they again tried to attribute the J-shaped association to the activation of the renin-angiotensin-aldosterone system (RAAS) and the sympathetic system due to salt reduction,^1^ but ignored the fact that these compensatory responses are only seen when the decrease in salt intake is large and sudden, e.g. from 20 to < 1 g/day for only a few days.^16^ When salt intake is reduced modestly over a prolonged period of time (which is the current public health recommendation), such responses are small.^17,18^ In addition, thiazide diuretics also stimulate the RAAS and, in the short term, the sympathetic nervous system. Yet, they do not increase mortality, but quite the opposite: in randomised trials, long-term treatment with thiazide diuretics significantly reduced CVD morbidity and mortality in hypertensive individuals.^19^ Moreover, given that both the relationship between salt intake and BP, and that between BP and CVD, are direct and continuous,^17^ it is hard to conceive how the association between salt intake and CVD risk could be J-shaped.

Another reason why the latest PURE results should be interpreted with particular caution is that, according to Mente et al, approximately three-quarters of the world’s population is eating harmfully low levels of salt and therefore should increase their intake! Furthermore, salt intake in several countries (e.g. Finland^20^ and the UK^21^) has been reduced to levels well below those that Mente et al claim are dangerous, and yet, the evidence clearly shows that such reductions have benefited public health.^22,23^ In England for instance, the average salt intake in 2003 was 9.5 g/day (as measured by 24-hour urinary sodium excretion in a random sample of the adult population). Following a successful salt reduction programme, salt intake was reduced to 8.1 g/day by 2011 (i.e. a reduction of 1.4 g/day in salt intake, which is equivalent to 0.6 g/day of sodium, and not the misquoted 0.4 g/day by Mente et al^1^). This resulted in a significant fall in population BP and mortality from stroke and ischaemic heart disease (Figure 2).^24^ Mente et al stated that ‘this finding was not observed in a later study’^1^:once again, they misquote their reference, the National Diet and Nutrition Survey, since the survey had never been set up to examine mortality.^25^ Cost-effectiveness analyses by the UK’s National Institute for Health and Care Excellence showed that salt reduction not only saves lives but also saves money: it estimated that in the UK, the salt reduction programme prevented around 9000 CVD deaths per year and saved around £1.5 billion in annual healthcare costs.^26^

**Figure 2.**
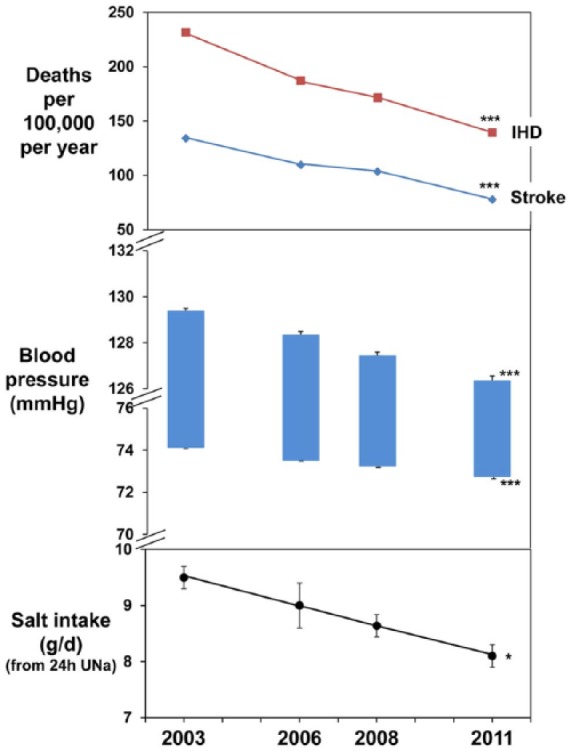
Changes in salt intake as measured by 24-hour urinary sodium excretion, blood pressure, stroke and ischaemic heart disease in England from 2003 to 2011. 24h UNa: 24-hour urinary sodium excretion; IHD: ischaemic heart disease.

Mente et al make a case for increasing potassium intake population-wide by consuming more fruits and vegetables.^1^ While we agree that this is a sound dietary measure, it requires individuals to make major changes in their eating habits. This is not the case with salt reduction, which can be achieved through reformulation, and is one of the most cost-effective, feasible and affordable measures to prevent CVD.^27^

Conducting rigorous, high-quality investigations of the relationship between salt intake and CVD is difficult. Unfortunately, the PURE study is not well designed for that challenge: unreliable salt intake estimations, lack of data points over time and susceptibility to residual confounding give way to statistical artefacts that are physiologically implausible, which large sample sizes and community-level analyses cannot improve. However large, studies with methodological limitations inevitably produce erroneous findings that cannot be corrected with statistics, and conclusions from such flawed studies should not be used to derail effective public health action.
